# An underestimated link: a study of eating disorders in patients followed for schizophrenia

**DOI:** 10.1192/j.eurpsy.2024.1531

**Published:** 2024-08-27

**Authors:** K. Razki, A. Larnaout, S. Ben Aissa, C. Najar, R. Lansari, W. Melki

**Affiliations:** Psychiatry department, Razi hospital, manouba, Tunisia

## Abstract

**Introduction:**

Schizophrenia is a debilitating mental illness that can cause significant disruptions in a person’s life, leading to difficulty with thinking, emotions, and behaviors. While the symptoms of schizophrenia are well-known and extensively studied, comorbidities like eating disorders are often overlooked and undertreated, despite their prevalence in patients with schizophrenia.

**Objectives:**

determine the different eating attitudes among schizophrenic patients and establish the link between eating attitudes, age, weight status, and psychotropic medication.

**Methods:**

This is a cross-sectional and descriptive study that took place from September to November 2022 among patients who consulted the post-care consultations of Psychiatry D service at Razi Hospital, Tunisia. We included patients who had been followed for at least one year for schizophrenia according to the diagnostic criteria of DSM-V and who had not relapsed for at least 2 months. The collection of sociodemographic and clinical data was done retrospectively by referring to the patients’ clinical records. Anthropometric measurements (weight, height, waist circumference, etc.) were recorded for each participant at the end of the interview. The Three-Factor Eating Questionnaire (TFEQ) was used to analyze eating attitudes.

**Results:**

According to our results among 30 patients followed for schizophrenia, 74% were men with a mean age of 45 years (3.8). Sixty percent of the participants had a BMI <18.5, 35% had a BMI between 18.5 and 25, and the rest had a BMI greater than 25. on the therapeutic level, 12 patients were on olanzapine, 15 patients were on risperidone and the rest were on haloperidol. The TFEQ score shows that uncontrolled eating was the most prevalent attitude in our population. A statistically positive association was found between uncontrolled eating and the use of olanzapine (p<0.05).

**Conclusions:**

Our study contributes to draw the attention of mental health professionals to the screening of eating disorders in patients followed for long term mental disorders and insists on multidisciplinary management to ensure a better quality of life for patients.

**Disclosure of Interest:**

None Declared

